# Expression of decoy receptor 3 in kidneys is associated with allograft survival after kidney transplant rejection

**DOI:** 10.1038/srep12769

**Published:** 2015-09-03

**Authors:** Shuo-Chun Weng, Kuo-Hsiung Shu, Ming-Ju Wu, Mei-Chin Wen, Shie-Liang Hsieh, Nien-Jung Chen, Der-Cherng Tarng

**Affiliations:** 1 Institute of Clinical Medicine, National Yang-Ming University, Taipei, Taiwan; 2 Center for Geriatrics and Gerontology, Taichung Veterans General Hospital, Taichung, Taiwan; 3 Division of Nephrology, Department of Internal Medicine, Taichung Veterans General Hospital, Taichung, Taiwan; 4 School of Medicine, Chung Shan Medical University, Taichung, Taiwan; 5 School of Medicine, College of Medicine, China Medical University, Taichung, Taiwan; 6 Department of Pathology, Taichung Veterans General Hospital, Taichung, Taiwan; 7 Genomics Research Center, Academia Sinica, Taipei, Taiwan; 8 Institute of Microbiology and Immunology, School of Life Sciences, National Yang-Ming University, Taipei, Taiwan; 9 Inflammation and Immunity Research Center, National Yang-Ming University, Taipei, Taiwan; 10 Department and Institute of Physiology, National Yang-Ming University, Taipei, Taiwan; 11 Division of Nephrology, Department of Medicine, Taipei Veterans General Hospital, Taipei, Taiwan

## Abstract

Decoy receptor 3 (DcR3) expression in kidneys has been shown to predict progression of chronic kidney disease. We prospectively investigated a cohort comprising 96 renal transplant recipients (RTRs) undergoing graft kidney biopsies. Computer-assisted quantitative immunohistochemical staining value of DcR3 in renal tubular epithelial cells (RTECs) was used to determine the predictive role of DcR3 in kidney disease progression. The primary end point was doubling of serum creatinine and/or graft failure. A multivariate Cox proportional hazards model was used to assess the risk of DcR3 expression in rejected kidney grafts toward the renal end point. In total, RTRs with kidney allograft rejection were evaluated and the median follow-up was 30.9 months. The greater expression of DcR3 immunoreactivity in RTECs was correlated with a higher rate of the histopathological concordance of acute T cell-mediated rejection. Compared with 65 non-progressors, 31 progressors had higher DcR3 expression (HDE) regardless of the traditional risk factors. Cox regression analysis showed HDE was significantly associated with the risk of renal end point with a hazard ratio of 3.19 (95% confidence interval, 1.40 to 7.27; P = 0.006) after adjusting for other variables. In repetitive biopsies, HDE in tissue showed rapid kidney disease progression due to persistent inflammation.

Both immunological and non-immunological risk factors contribute to long-term kidney allograft survival. The demographics and comorbidities of donors and recipients change continuously and thus it is necessary to develop precise models for prediction of allograft outcome. The optimal organ allocation system was used initially to determine factors associated with graft failure^1^,^2^. Subsequently, well-known molecules, such as transcription factor forkhead box P3 (FOXP3), mast cell transcripts, damage-associated molecular patterns (DAMPS), and complement activation have been proposed to be correlated with allograft rejection or scarring^3^,^4^,^5^,^6^. To date, very few markers of potentially modifiable disease have been identified. Therefore, new tissue biomarkers are needed to identify kidney transplant patients at higher risk for graft dysfunction and/or loss.

Decoy receptor 3 (DcR3) is a member of the tumor necrosis factor receptor (TNFR) superfamily, but it lacks the transmembrane domain as a secreted protein^7^,^8^. Investigators indicated that overexpression of DcR3 in cancer cells predicted poor survival in patients with gastrointestinal tract tumors^9^,^10^. DcR3 is not expressed in normal human kidney tissues^7^, but serum DcR3 levels are higher in patients with chronic kidney disease (CKD) as compared with those in cancer patients or normal individuals^11^. Our previous study identified DcR3 expression in renal tubular epithelial cells (RTECs) of the renal cortex as a novel biomarker for progression in CKD patients^12^. Overexpression of DcR3 has been linked in part to renal fibrogenesis through its blocking of Fas-induced apoptosis of myofibroblasts^12^,^13^. However, DcR3 has been recently reported to ameliorate the development of autoimmune crescentic glomerulonephritis (ACGN) through immunosuppression in a mouse model^14^. Human DcR3 (hDcR3) decreased the diffuse infiltration of T cells, monocytes/macrophages, and proinflammatory cytokines in the ACGN mouse model, but the hDcR3 level in serum was extremely high in this animal model, exceeding the range of DcR3 in healthy subjects and CKD patients^11^,^14^.

Soluble DcR3 has emerged as a pleiotropic immunomodulator which is immune-evasive and able to promote type 2 T helper cells (Th2) in organ transplantation. Indeed, a very high dose of DcR3-Fc can suppress alloantigen-stimulated mouse T cell activation and inhibit cytotoxic T lymphocyte development^8^,^15^,^16^. But, with *in situ* hybridization of human kidney tissue, RTECs up-regulated TNFR-2 mRNA, which is characteristic of allograft rejection^17^, whereas signaling through these receptors is complex and not well understood. To date, the effects of DcR3 (TNFR superfamily 6B) on human kidney allograft rejection and survival remain unclear. In this study, we hypothesized that DcR3 reflects persistent rejection and insidious inflammation, while it is up-regulated. Therefore, our aim was to investigate whether DcR3 would be expressed in the kidneys of patients with allograft rejection and if so, such expression could be a tissue biomarker for prediction of disease progression after acute allograft rejection.

## Results

**Figure 1:**
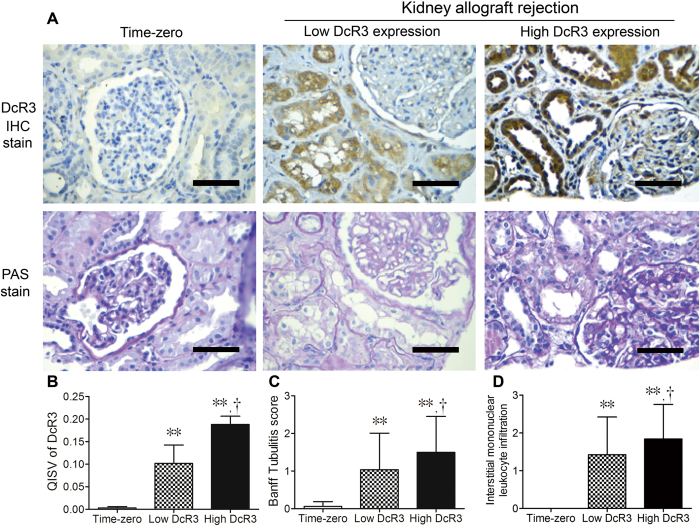
Representative photographs of immunohistochemical (IHC) staining of decoy receptor 3 (DcR3) and periodic acid-Schiff (PAS) staining in kidney allograft rejection and time-zero biopsy. (**A**) The IHC staining of DcR3 and PAS staining. (**B**) The quantitative immunohistochemical staining value (QISV) of DcR3 was assessed by computer-assisted quantitative analysis. (**C**) Banff tubulitis scores and (**D**) Banff interstitial mononuclear leukocyte infiltration were assessed afterward under PAS staining by a pathologist. Data are expressed as means ± standard deviation. *P < 0.05 high or low DcR3 expression *vs* time-zero biopsies. ^†^P < 0.05 high DcR3 expression *vs* low DcR3 expression. Scale bar, 50 μm.

In time-zero biopsy samples without acute tubular injury that served as the controls, DcR3 immunoreactivity was undetectable (Fig. 1A). Among patients with transplant rejection, DcR3 staining was predominantly in the RTECs of the renal cortex, not in the glomeruli, interstitium, or vessels in severe rejection kidney (Fig. 1A). The preferential staining in rejection kidney was both proximal and distal renal tubules (Figure S1).

### Baseline characteristics of patients

The median optimal quantitative immunohistochemical staining value (QISV) of DcR3 was 0.156 for identifying the primary end point and the cutoff value (QISV = 0.156) was validated by the area under the ROC curve (AUC: 0.686, 95% CI 0.571 to 0.800, P = 0.003) for predictive accuracy. The high DcR3 expression (HDE) and low DcR3 expression (LDE) groups did not differ significantly with respect to age, gender, and risk factors associated with prognosis of kidney disease (Table 1). There were no differences in recipient risk score^2^,^18^,^19^ assessed by the recipient’s age, diabetes mellitus status, ischemic heart disease status, and duration of dialysis between the two groups. The HDE group had a lower donor risk score (P = 0.022; Table 1)^2^,^18^,^19^ assessed by the donor’s age, history of hypertension, eGFR, number of HLA mismatches, and cause of death. eGFR was calculated by the 4-variable modification of diet in renal disease (MDRD) formula^20^. Urinary protein excretion rate, percentage of fibrosis in renal interstitium, antihypertensive drugs, antiproliferative regimen, and calcineurin inhibitors showed no significant differences between the two groups. Pathogenesis-based immunopositivity of DcR3 revealed the major finding that there were more cases of acute rejection in the HDE group than in the LDE group (70.5% *vs* 42.3%; P = 0.006), especially in the subgroup of acute T cell-mediated rejection (TCMR).

### HDE correlated with acute TCMR manifesting tubulitis and interstitial inflammation

In time-zero biopsies, we observed normal cellularity in the glomeruli and interstitium; partially intact brush borders and retainable basement membranes in the glomeruli and tubules in PAS staining (Fig. 1A). Slightly less stained time-zero biopsies showed DcR3 had low QISV (0.003 ± 0.003). The QISVs of DcR3 in the RTECs were 0.188 ± 0.018 in the HDE group and 0.102 ± 0.041 in the LDE group (P < 0.001; Fig. 1B), respectively. Compared with the LDE group, there was a gradual increase in loss of tubular cell brush borders, greater variation in cell size and shape and patchy desquamation of individual epithelial cells, leaving bare basement membranes in the HDE group in PAS staining (Fig. 1A). Tubulitis (t-score) and interstitial mononuclear leukocyte infiltration (i-score) were significantly higher in the HDE group than in the LDE group (t-score: 1.50 ± 0.95 *vs* 1.04 ± 0.97; P = 0.013; i-score: 1.81 ± 0.95 *vs* 1.44 ± 0.97; P = 0.033) (Fig. 1C,D). Glomerular obsolescence, glomerulitis, and intima arteritis were similar for both groups. Given that the HDE group showed more pronounced tubular injury than the LDE group, we also tested the DcR3 immunohistochemical staining and PAS on biopsy without rejection or with borderline infiltrates (Figure S2).

The DcR3 expression was more specifically related to the severity of acute TCMR (Figures S3A and S3B). In the subgroup analysis, Figures S3C and S3E showed positive correlation between HDE and histologic features of acute TCMR, such as tubulitis (HDE *vs* LDE; P = 0.037) and interstitial mononuclear leukocyte infiltration (HDE *vs* LDE; P = 0.010). Although there were cases of high DcR3 in the acute ABMR group, the relationship between HDE and pathologic representation (peritubular capillaritis, glomerulitis, and complement 4d (C4d) staining by immunohistochemistry (IHC, data not shown)) of acute ABMR was not statistically significant (Figures S3D and S3F).

### HDE independently predicted poor graft outcome

With regard to the primary end point, 6 patients (5.3%) had a two-fold increase in serum creatinine, 20 patients (17.7%) had reached end-stage renal failure requiring renal replacement therapy, and 5 patients (4.4%) died with deteriorated renal function. Other independent associations with worse graft function were found, including low serum albumin (P = 0.001), increased proteinuria (P = 0.004), low eGFR (P < 0.001), severe interstitial fibrosis/tubular atrophy (IF/TA) (P = 0.006), high chronic allograft damage index (CADI, P = 0.008), and C4d staining by IHC (P = 0.049) at the time of biopsies (Table 2).

**Figure 2:**
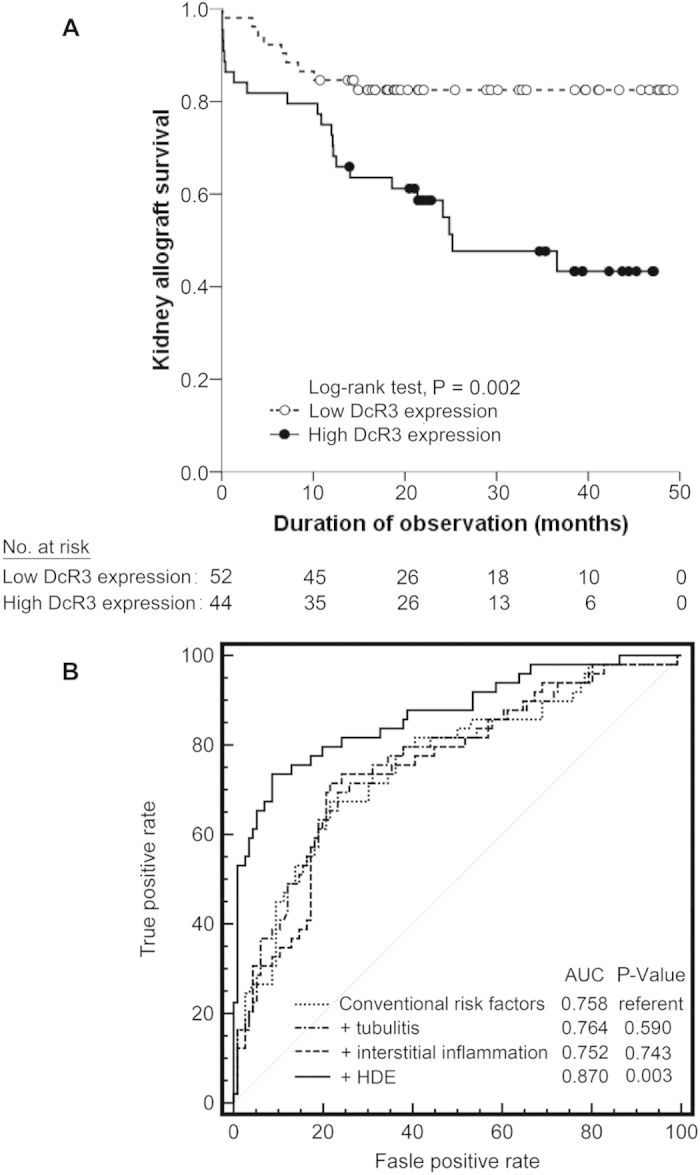
Survival curves and receiver operating characteristic (ROC) curve for predictability of outcome in renal transplant recipients with high and low DcR3 expression. (**A**) Kaplan-Meier cumulative curves for end points of the composite: doubling of serum creatinine or graft failure. (**B**) Risk prediction was assessed by the ROC curve. Each biomarker was added stepwise to the model of conventional risk factors to assess the AUC change for predicting progression of kidney disease in allografts. Conventional risk factors included age, sex, hypoalbuminemia, eGFR, proteinuria and chronic allograft damage index (CADI).

The Kaplan-Meier survival analysis revealed that 9 of 52 cases of LDE and 22 of 44 cases of HDE progressed to the primary end point (Log-rank test, P = 0.002; Fig. 2A). Patients with HDE had worse renal survival and significantly shorter progression time compared with LDE patients: mean times to progression were 27.9 (95% CI, 22.1 to 33.7) months compared with 41.8 (95% CI, 37.3 to 46.2) months (P = 0.002), respectively.

In age- and gender-adjusted Cox regression analysis (Table 3, model 1), HDE, hypoalbuminemia, proteinuria, lower eGFR, C4d staining by IHC, intimal or transmural arteritis, relatively severe IF/TA (26–50% + > 50%), and CADI were strongly associated with progression to renal end point (all P < 0.05). Multivariate Cox regression analysis (Table 3, model 2) showed that HDE in RTECs independently had a higher risk of kidney disease progression with an HR of 3.19 (95% CI, 1.40 to 7.27; P = 0.006) by adjusting age, gender and other significant variables.

### HDE significantly increased the predictability of kidney disease progression

Risk prediction was assessed by incremental change in the area under the ROC curve (AUC) (Fig. 2B). To test discrimination ability, the classification of QISVs of DcR3 was incorporated into the group of conventional risk factors (base model) for predicting the primary end point. The AUC of the base model was 0.758. Traditional risk factors was included in the base model^14^,^16^. The Banff tubulitis and interstitial inflammation scores showed no increase in testing the predictive value of the combined outcome. However, the addition of high DcR3 expression, a molecular classifier, to the group of conventional risk factors further improved the predictability of a model for kidney disease progression (AUC 0.870; P = 0.003).

### HDE was a biomarker of persistent insidious inflammation and further fibrosis in the repetitive allograft biopsy

**Figure 3:**
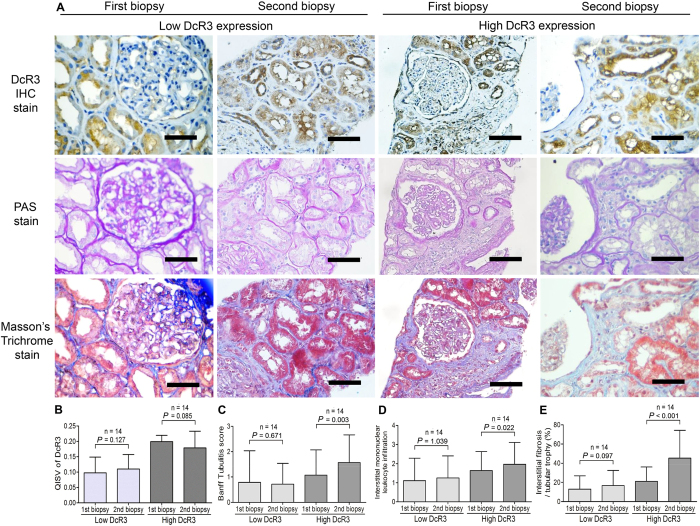
Representative photographs and quantitative values of repetitive biopsy data of immunohistochemical (IHC) staining of DcR3, periodic acid-Schiff (PAS), and Masson’s trichrome staining in kidney allograft rejection. (**A**) The IHC staining of DcR3, PAS, and Masson’s trichrome staining. (**B**) In repetitive biopsies, the QISV of DcR3 in the HDE group maintained a significantly higher level than that in the LDE group. (**C**) Tubulitis significantly increased in the HDE group in repetitive biopsies (P = 0.003). (**D**) Interstitial mononuclear leukocyte infiltration significantly increased in the HDE group in repetitive biopsies (P = 0.022). (**E**) The interstitial fibrosis and tubular atrophy scores were compared in the repetitive biopsies in the LDE (P = 0.097) and HDE groups (P < 0.001). There was no difference in time-dependent effect between the 1^st^ and 2^nd^ biopsies in the LDE and HDE groups. Scale bar: 50 μm.

The 28 cases with diagnoses of acute rejection and repetitive biopsy during the study interval provide strong evidence that the biomarker HDE can serve as a good prognosis indicator (Fig. 3A). In the HDE group, the QISV of DcR3 was high in the repetitive follow-up biopsies, and Banff tubulitis (P = 0.003) and interstitial mononuclear leukocyte infiltration (P = 0.022) were also more severe (Fig. 3B–D). Moreover, IF/TA was significantly increased in the repetitive biopsies of the HDE group (n = 14; P < 0.001; Fig. 3E).

### Immunofluorescence double staining and confocal microscopy

**Figure 4:**
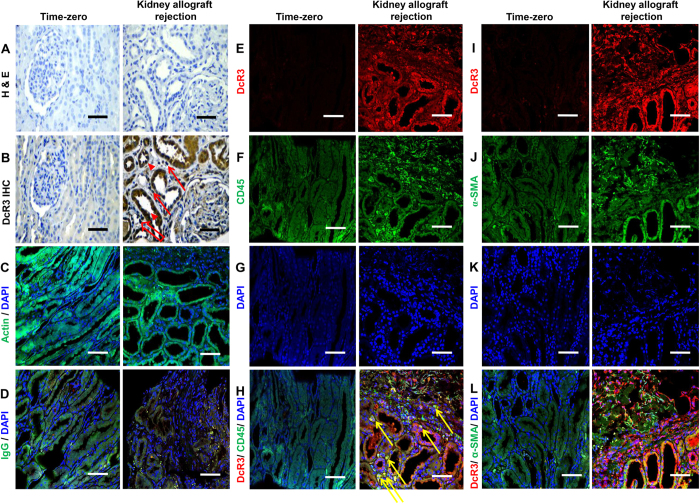
Representative photographs of immunohistochemical (IHC) staining and immunofluorescence (IF) double staining between severe kidney allograft rejection and time-zero biopsies. About IHC, the samples were immunolabeled with mouse anti-hDcR3, visualized using anti-mouse horseradish peroxidase (HRP) and mounted in high-sensitivity diaminobenzidine (DAB) chromogenic substrate. (**A**) Negative controls showed no immunostaining of decoy receptor 3 (DcR3). (**B**) Representative images show that DcR3 was not expressed in time-zero biopsies of kidney tissue of positive control subjects. In mixed-type acute rejection, acute T cell-mediated rejection was composed of Banff Type IIA [i-Banff: 2 (red arrow), t-score: 2 (red arrowhead), v-score: 1)]; in acute antibody-mediated rejection, type II [peritubular capillaritis (red hollow arrow)] showed strong DcR3 expression in RTECs. About IF double staining, the second antibodies (anti-rabbit IgG-FITC with green fluorescence and anti-mouse IgG-PE with red fluorescence) is for immunoreactivity. (**C**) Monoclonal anti-β-Actin antibody as internal control is for confirming technique in immunofluorescence staining. (**D**) The isotype antibody IgG is for negative control in the double immunofluorescence staining. (**E**) High DcR3 expression (HDE) was observed in the renal tubules and infiltrating mononuclear leukocytes in severe kidney rejection allograft. (**F**,**J**) CD45 and α-smooth muscle actin (α-SMA) were presented with green fluorescence in leukocyte cell membrane and interstitial myofobroblasts, respectively. (**G**,**K**) 4′-6-Diamidino-2-phenylindole (DAPI) is for nucleus. (**H**) There is colocalization between DcR3 and CD45 in severe kidney rejection allograft (yellow arrow). (**I**) HDE was obvious in the atrophic tubules of kidney rejection allograft. (**L**) No colocalization between DcR3 and α-SMA. Scale bar, 50 μm.

There is obvious colocalization between common leukocyte antigen (CD45 surface marker) and DcR3 expression over infiltrating mononuclear leukocytes in the renal tubules, renal interstitium, and peritubular capillaries (Fig. 4H). The opposite situation (no colocalization) is found between interstitial myofibroblasts (α-smooth muscle actin, α-SMA) and DcR3 expression (Fig. 4L), even in the severe kidney rejection allograft. But in the near interstitial fibrosis and tubular atrophy area, there is also high expression of DcR3 in the residual tubules (Fig. 4I).

### *In Situ* Hybridization

In the areas of active inflammation and severe rejection-related architecture, there is intense DcR3 mRNA expression in RTECs infiltrated with mononuclear cells (Figure S4B). The pattern seems to be like DcR3 IHC staining (Figure S4A), and it appears endogeneous DcR3 is locally produced by mononuclear cells and RTECs.

## Discussion

To the best of our knowledge, this is the first study to demonstrate that DcR3 is significantly expressed in tissues of kidney transplants with acute rejection. We also find that in cases with TCMR, increased expression of DcR3 is correlated with histopathology of Banff i-scores and t-scores. To begin with, the association of HDE with tubulitis and interstitial monocyte infiltration may lead to renal fibrosis and subsequent loss of graft function in RTRs with acute rejection. In addition, the high expression of DcR3 molecules in RTECs is associated with worse prognosis compared to that of allografts with low DcR3 expression. Events are defined as either allograft loss with return to dialysis or persistent (>3 months) doubling of serum creatinine. Intriguingly, the predictive value of HDE in RTECs increases the statistical power for outcome prediction calculated by traditional risk factors.

DcR3 is a soluble decoy receptor and member of the TNFR superfamily; it is up-regulated following cell injury and/or inflammation caused by acute rejection^8^,^17^,^21^,^22^. Recent evidence has supported the first-hit theory that DcR3 overexpression in damaged tubuli correlates with acute TCMR under an immediately inflammatory cytokine storm, such as that generated by TNF-α or IL-1β, which enhances DcR3 expression via a nuclear factor-κB (NF-κB) dependent pathway^22^,^23^. The DcR3 level was found to significantly increase in the culture supernatant of TNF-α-stimulated HK-2 cells, a human proximal tubular epithelial cell line in a previous study^12^ and the current one. Furthermore, the TNF-mediated DcR3 induction in HK-2 cells could be down-regulated by treating the cells with inhibitors of the MAPK kinase signaling pathway and NF-κB pathway (Figure S5). These observations supported the implication that, in the rejected kidney, DcR3 could be induced locally from RTECs during inflammation. The histology clearly shows a tubular expression pattern which seems to be most intense DcR3 mRNA expression in damaged tubuli (Figure S4B). In this prospective study, DcR3 really has immunological effects during acute T cell-mediated rejection (Fig. 4H). Of note, in several inflammatory diseases, DcR3 can directly induces NF-κB–mediated expression of adhesion molecules and inflammatory cytokines by monocytes^22^,^23^,^24^,^25^. In the stage of acute cellular immunologic storm, there is possibility of staining on the leukocyte subsets which is proved by DcR3 expression and their surface markers, respectively (Fig. 4H). In a clinical entity, DcR3 expression in proximal and distal tubules reflects an effector molecule during acute rejection. But in the fibrotic tissue, the role of DcR3 in the RTECs which showed high was to prevent apoptosis (Fig. 4I). Locally expressed DcR3 in the RTECs may suppress the FasL-Fas-mediated apoptosis^15^,^17^, leading to an accumulation of activated allo-responding T cells, which consequently aggravates the tissue inflammatory response in allograft rejection. Increased local DcR3 may also serve as a negative feedback modulator to interfere with the interaction between LIGHT and its receptors, which mediates the co-stimulation and activation of allo-responding T cells^26^.

The second-hit would be caused by subsequent residual inflammation and would promote renal fibrosis (Fig. 3). Based on human serum levels in acute rejection patients (Figure S6), serum DcR3 level was relatively low as compared to the levels in mice treated with hDcR3 or transgenic overexpression (150 to 850 ng/mL)^14^,^27^. The endogenous DcR3 expression may not be high enough to cope with the modulation of the T-cell response. However, as shown in our previous CKD study, HDE in the damaged tubuli could annotate peripheral myofibroblast escaping from Fas and FasL-induced apoptosis^12^.

Endogenous tissue DcR3, which has the characteristic of a molecular classifier, is suitable for consideration as a novel biomarker for acute TCMR. However, HDE, the molecular phenotype, might be associated with future graft dysfunction. The representative HDE in RTECs is better than Banff criteria available now for acute T cell rejection - tubulitis and interstitial mononuclear leukocyte infiltration. This means that the cumulative burden of injury is more correlated with functional disturbance and risk of future graft loss in both living-related and suboptimal grafts. The worsening of tubulitis and interstitial inflammation in cases with HDE suggests that HDE may identify lesions of acute TCMR in particular patients that do not respond to anti-rejection therapy (Table S1) and may be a surrogate marker of failure of treatment response which is well documented to be associated with allograft failure.

Matching recipient group to donor grade as proposed by the United Network for Organ Sharing (UNOS) will improve renal survival^1^,^2^,^18^,^19^. Banff working groups have continuously revised a clinically relevant morphological classification and developed available immunobiological and immunopathology sessions^28^. CADI is useful to quantify renal allograft histology and has demonstrated the relative prediction of graft outcome^29^. Donor graft qualities as well as non-immunological factors in RTRs, such as hypertension, dyslipidemia, and ischemia-reperfusion injury are associated with increased risk of progression of renal allograft damage. The correlation of increased expression of DcR3 with the pathological stage of acute TCMR supports the notion that this molecule is not simply a mediator in transplant immunology. Furthermore, IF/TA significantly increases in the repetitive biopsies of the HDE group, indicating that expression of endogenous DcR3 also facilitates the development of renal fibrosis in cases with repetitive rejections^12^,^13^.

Some limitations in this study should be acknowledged. First, our study is prospective and observational in nature, so it cannot prove causality. Second, the IHC staining is described as the computer-assisted QISV value, and it is at best quantitative for the antigen target^21^,^30^. Better impression could be gained by assessment not of pixels but of morphological structures i.e. percentage of tubuli affected. However, we have tried our best to standardize the whole staining and categorical procedures. Something we can’t substitute is the quantification method. About the immunohistochemical staining, we just used pixel intensity. But in the other Banff pathological scoring, we used the morphologic structures. Despite the obvious finding of DcR3 expression in renal allografts, further study should be conducted in protocol biopsies to clarify whether DcR3 is only a nonspecific inflammatory marker or is up-regulated during acute T cell-mediated rejection (TCMR).

In conclusion, we found the overexpression of DcR3 in tissue to be a novel biomarker which correlates with acute TCMR. When the DcR3 molecular classifier is used in combination with pathologic findings and traditional risk factors, it improves prediction of kidney disease progression in RTRs. Further study is needed to explore the possible therapeutic potential or detrimental effects of DcR3 in kidney graft outcome.

## Methods

### Study design and participants

Patients were eligible for enrollment if they underwent transplant biopsy for clinical indications such as deterioration in kidney function, proteinuria, and overt kidney allograft dysfunction at Taichung Veterans General Hospital between September 1, 2010 and November 15, 2013. Criteria for inclusion were age >20 years, first kidney transplantation from an inshore deceased or living donor, kidney injury with different spectrum of rejection, mostly acute rejection, and repetitive biopsies. Among the 168 patients who consented to undergo graft kidney biopsy, 18 (10.7%) cases were excluded due to refusal to sign the informed consent document, pure severe acute tubular necrosis (ATN) without kidney rejection, and recurrent glomerulonephritis after kidney transplantation, as well as active malignancy. Sixteen (9.5%) cases were excluded due to non-rejection caused by hemodynamic change, arteriosclerosis or donor conditions, and polyomavirus nephropathy. Thirty-eight (22.6%) cases with repetitive allograft biopsy having first biopsy were included, but the second biopsy was initially excluded. Ninety-six patients who met the inclusion criteria which we focused on kidney rejection^31^ were enrolled in the study. The prospective cohort was assessed for more than 6 months in order to prevent lead-time bias. The primary study end point was a composite: doubling of serum creatinine or return to dialysis following graft failure or death with deteriorated kidney function^12^,^32^,^33^. The control group comprised five subjects who received time-zero graft kidney biopsy after revascularization which showed kidney injury that was not acute. The study was approved by the Institutional Review Board of Taichung Veterans General Hospital. Written informed consent was obtained from each of the enrolled study patients and control subjects. The clinical and research activities being reported are consistent with the Principles of the *Declaration of Istanbul* as outlined in the ‘*Declaration of Istanbul on Organ Trafficking and Transplant Tourism*’.

### Histopathology and immunohistochemistry

Kidney specimens were fixed in formalin then immersed in phosphate buffer saline (PBS). After dehydration with a graduated series of ethanol and xylene, the tissues were embedded in paraffin and cut into 4-μm sections. Normal kidney allograft tissues of the 5 control subjects were also processed. After deparaffinization and rehydration, hematoxylin and eosin stain, periodic acid-Schiff (PAS) stain, and Masson’s trichrome stain were used for histological examination, Banff 2009 classification^34^, and determination of IF/TA, respectively. PAS stain included periodic acid solution (SigmaUltra), Schiff reagent (Sigma-Aldrich), sodium bisulfite solution (Sigma-Aldrich), and hematoxylin. Masson’s trichrome kit (Accustain, Sigma-Aldrich, St. Louis, MO, USA) was used according to the manufacturer’s instructions.

Standard immunohistochemical (IHC) protocol was also followed after deparaffinization and rehydration. Then, each 4-μm section in 10 mM sodium citrate (pH 6.0) was heated in a microwave oven (650W, 12min) for antigen retrieval, after which endogenous peroxidase activity was blocked by 3% hydrogen peroxide. Thereafter, adjacent sections from the same paraffin block were incubated with the primary antibodies: mouse monoclonal antibodies directed against human DcR3 (diluted 1:20; BioLegend, San Diego, CA, USA) at 4 °C overnight. Then, all tissue sections were incubated with secondary antibody (Envision + Dual Link System – Horseradish Peroxidase [HRP], DakoCytomation) for 30 minutes at room temperature. Signals were developed with two-component high-sensitivity diaminobenzidine (DAB) chromogenic substrate (DakoCytomation) for 10 minutes and counterstained with hematoxylin. For negative controls, the primary antibodies were replaced by an equal concentration of isotype-matched irrelevant antibodies (DakoCytomation). An adjacent paraffin section of the slide was used as the negative control (Fig. 4A,B).

### Quantification of histopathology and immunohistochemistry

For all histopathological findings, the final pathological diagnosis and semiquantitative analysis were conducted according to the Banff 2009 classification^34^. The percentage of global obsolescence of glomeruli and the severity of tubulointerstitial injury were examined under 20 randomly selected high-power fields (×400). The IF/TA score was calculated under Masson’s trichrome stain using a 20× objective. All sections were assessed afterward by a pathologist (Mei-Chin Wen), who was blinded to the clinical outcomes and laboratory data.

An evaluation of cortical tubulointerstitial IHC staining for DcR3 as determined by computer-assisted pixel counts (Image-Pro Plus 6.0, Media Cybernetics, Silver Spring, MD, USA) was performed for potential correlation with histologic and outcome parameters. Twenty areas from each IHC sample were randomly selected in the renal cortex and examined under a microscope. Briefly, the selected non-overlapping high-power fields of each section were captured by a DM750 microscope (Leica, Wetzlar, Germany), and then the images were converted to digital files using a CCD camera (Nikon, COOLPIX, P6000, Tokyo, Japan). The medulla, glomeruli in the cortex, and the large vessels were eliminated using Adobe Photoshop CS6 to specifically measure the DcR3 expression in the RTECs. Before performing the computer-aided quantitative staining analysis, the intensity in the blank area of slides was used for calibration of optical density. We used two color segmentations: one recognized DcR3-positive brown cells and the other DcR3-negative blue background cells. The integrated optical density (IOD) was obtained as the total number of immunopositive brown pixels multiplied by the brown intensity of those pixels, and it was used to avoid the data simply being a correlated metric of inflammatory infiltration^30^. QISV was calculated as the IOD divided by the total area occupied by the brown and blue cells (Figure S7)^12^,^35^.

### Immunofluorescence double staining and confocal microscopy

There are mildly stained nuclei of tubulointerstitial infiltration and mononuclear leukocytes with DcR3 in rejection kidneys. To realize the colocalization of DcR3 (diluted 1:15; anti-mouse antibody; BioLegend, San Diego, CA, USA) and common leukocyte antigen (diluted 1:250; CD45 antibody, anti-rabbit antibody; Santacruz, H-230: sc-25590, Europe), we used paraffin sections following the double stain protocol^36^. The same procedure was done to see whether DcR3 and interstitial myofibroblasts (diluted 1:200; α-SMA antibody, anti-rabbit antibody; Abcam: ab5694, USA) colocalize in the same site. The second antibodies (anti-rabbit IgG-FITC) with green fluorescence was used for CD45 and α-SMA with wavelength of 647 nm (Sigma-Aldrich). The red fluorescence (Anti-mouse IgG-PE) for DcR3 expression with wavelength of 488nm (Sigma-Aldrich) was used. Anti-βactin antibody [diluted 1:5000; anti-mouse IgG (Fab)-FITC, Sigma-Aldrich, A5441] with green fluorescence served as an internal control, and those primary antibodies stayed overnight. Isotype IgG was used as an negative control. 4′-6-Diamidino-2-phenylindole (DAPI) could transmit cell membrane and bind the double strand DNA in the nucleus with wavelength of 358 nm (blue). Then, we used an optical imaging technique for increasing optical resolution and contrast of a micrograph by the confocal microscopy (confocal laser scanning microscope, Olympus FV1000, Japan).

### *In Situ* Hybridization

Paraffin embedded sections were dewaxed and rehydrated, then the tissue was digested with pepsin 1 μg/mL in 0.1 N HCl (diluted in RNase-free water) for 30 mins at 37 °C and acetylated in freshly prepared 1:10 triethanolamine with MgCl_2_, pH 8 and 0.25% acetic anhydrite. Tissues were prehybridized for 2 hrs at 48 °C in a hybridization buffer containing 50% formamide, 5 × SSC, RNase inhibitor and 10 μg/ml Salmon sperm DNA (sssDNA, Sigma-Aldrich). The above procedures were used to expose nucleic acids. Decoy receptor 3 cDNA plasmid was earlier transformed into *Escherichia coli*, and it was extracted. Then this plasmid was digested with KpnI, which brought about the 650 bps cDNA template for further probe production. The inserted human DcR3 cDNA was flanked by SP6 and T7 sites for RNA polymerase (Roche, Germany). The cDNA (288 bps between nts 446 ~ 733; Genbank #AF104419) was designed to provide high sensitivity for *in situ* hybridization of DcR3 mRNA from the gene^37^. The preparation of probe detection was an *in vitro* transcription with sense (negative control) and anti-sense the SP6/T7-Polymerase Digoxigenin (DIG)-Labeling and Transcription Kit (Roche, Germany). The DIG-labeling probe was then added on the slides in new hybridization buffer, denatured for 10 mins at 65 °C and incubated overnight at 45 °C. The slides were washed 20 mins in 2 × SSC, 15 mins in 1 × SSC (twice), 15 mins in 0.2 × SSC at 56 °C and 15 mins in 0.2 × SSC at room temperature. After blocking (fetal bovine serum, FBS) for 1 hr, slides were then incubated for 2 hrs at 37 °C with a monoclonal anti-digoxin-alkaline phosphatase antibody (Anti-digoxigenin) produced in mouse (Sigma-aldrich) and subsequently washed in a Tris pH 7.5-NaCl buffer. Color was developed for signal detection with NBT/BCIP (Roche, Germany).

### Statistical Analysis

Categorical variables were expressed as numbers and percentages using the Pearson χ^2^ test, but for an expected count of less than 5, Fisher’s exact test was used. The Kolmogorov-Smirnov test was used to test whether the continuous variables of the sample had a normal distribution. Continuous variables are expressed as means ± SD or medians and interquartile ranges. For between-two-group comparisons, the Student’s *t*-test was used for normally distributed data and the Mann-Whitney rank sum test was used for data with a non-normal distribution. A cumulative survival curve of the kidney allograft was generated by the Kaplan-Meier method, and the high and low expressions of DcR3 in the RTECs were compared by the log-rank test. The Cox proportional hazard models are presented as hazard ratios (HR) and 95% confidence intervals (CI). Multivariate Cox regression analysis was used to examine the association between DcR3 expression and the primary end point, with adjustment for potential confounding factors. All demographic variables with a hazard ratio were adjusted for age and gender with a P value < 0.05 (Table 3, model 1). The established risk factors, such as age, gender, hypoalbuminemia, urine protein excretion rate, estimated glomerular filtration rate (eGFR), IF/TA, and chronic allograft damage index (CADI)^29^ were considered to be potential confounders (Table 3, model 2). The individual features of acute rejection and chronic lesions were taken into account in the multivariate analysis to determine whether DcR3 is a better independent predictor of outcome than the microscopic findings that we already know are important^28^. To assess discrimination ability, area under the receiver operating characteristic (ROC) curves were used for models of conventional risk scores, followed by stepwise addition of Banff tubulitis or interstitial inflammation scores to high QISV of DcR3 expression, which was calculated for prediction of kidney disease progression^12^,^24^,^38^. All statistical tests were carried out with the Statistical Package for the Social Sciences (version 15.1; SPSS, Inc., Chicago, IL, USA) and MedCalc software (1993-2008, Frank Schoonjans, the Netherlands).

## Additional Information

**How to cite this article**: Weng, S.-C. *et al*. Expression of decoy receptor 3 in kidneys is associated with allograft survival after kidney transplant rejection. *Sci. Rep*. **5**, 12769; doi: 10.1038/srep12769 (2015).

## Supplementary Material

Supplementary Information

## Figures and Tables

**Table 1:** Characteristics of renal transplant recipients with high and low decoy receptor 3 (DcR3) expressions.

Characteristic	Low DcR3 expression	High DcR3 expression	P Value
	(n = 52)	(n = 44)	
Demographics			
Age at biopsy (yr)	49.7 ± 12.0	50.1 ± 12.2	0.870^a^
Men (n [%])	28 (53.8)	19 (43.2)	0.298^b^
Diabetes mellitus (n [%])^e^	16 (30.8)	13 (29.5)	0.896^b^
Systolic BP (mmHg)	134.6 ± 19.7	140.1 ± 20.6	0.185^a^
Body mass index (kg/m^2^)	22.9 ± 3.2	24.5 ± 4.8	0.063^a^
Timing of the biopsies (months; median [interquartile range])	81.9 (14.3–180.6)	56.1 (6.7–123.7)	0.162^d^
Total HLA mismatches	3.2 ± 1.3	2.7 ± 1.3	0.072^a^
PRA class I ≥ 10% (n [%])	6 (11.5)	6 (13.6)	0.757^b^
PRA class II ≥ 10% (n [%])	8 (15.4)	4 (9.1)	0.353^b^
Previous acute rejection (n [%])	13 (25.0)	13 (29.5)	0.618^b^
Recipient risk score (A–D, 4 grades)	1.5 ± 0.6	1.6 ± 0.7	0.382^a^
Donor age (yr)	32.6 ± 12.2	29.4 ± 7.1	0.109^a^
Live donor (n [%])	16 (30.8)	12 (27.3)	0.707^b^
Cold ischemia time (hours)	8.9 ± 4.3	8.1 ± 4.3	0.321^a^
Donor risk score (0–39 points)	9.0 ± 6.8	6.3 ± 4.6	0.022^a^
Laboratory data			
Serum albumin (g/dL)	3.8 ± 0.5	3.7 ± 0.6	0.348^a^
Total cholesterol (mg/dL)	197.9 ± 49.1	197.3 ± 54.2	0.955^a^
Urine protein (g/24 h; median [interquartile range])	0.49 (0.18–1.93)	0.55 (0.30–1.88)	0.506^d^
eGFR, MDRD (ml/min/1.73 m^2^)	31.3 ± 15.4	27.8 ± 15.5	0.273^a^
Histopathology of renal allograft biopsy			
Acute rejection (n [%])	22 (42.3)	31 (70.5)	0.006^b^
TCMR	14 (26.9)	22 (50.0)	
ABMR	5 (9.6)	5 (11.4)	
Mixed TCMR and ABMR	3 (5.8)	4 (9.1)	
Borderline rejection (n [%])	14 (26.9)	10 (22.7)	0.636^b^
CAMR and Transplant glomerulopathy (n [%])	16 (30.8)	3 (6.8)	0.004^c^
IF/TA (n [%])			0.458^b^
0	12 (23.1)	13 (29.5)	
<25%	28 (53.8)	18 (40.9)	
26–50%	7 (13.5)	10 (22.7)	
>50%	5 (9.6)	3 (6.8)	
CADI scores (0–18 points)^f^	6.0 ± 3.6	5.9 ± 3.3	0.911^a^
Medications			
ACEI/ARB (n [%])	28 (53.8)	15 (34.1)	0.052^b^
Immunosuppression (n [%])			0.096^b^
CNI + MMF-based	48 (92.3)	33 (75.0)	
CNI + mTOR inhibitor-based	2 (3.8)	3 (6.8)	
mTOR inhibitor-based	0 (0.0)	2 (4.5)	
Other	2 (3.8)	6 (13.6)	

Comparison by

^a^independent-samples *t* test.

^b^Chi-square test.

^c^Fisher’s Exact test and.

^d^Mann-Whitney test with IQR, interquartile range. HLA, human leukocyte antigen (Total HLA mismatches at HLA-A, -B, -DR loci); MDRD, Modification of Diet in Renal Disease formula; PRA, panel-reactive antibodies; TCMR, T cell-mediated rejection; ABMR, antibody-mediated rejection; CAMR, chronic active antibody-mediated rejection; CAN, chronic allograft nephropathy; IF/TA, interstitial fibrosis and tubular atrophy; CADI, chronic allograft damage index; ACEI / ARB: angiotensin converting enzyme inhibitor/angiotensin-II receptor blocker; CNI, calcineurin inhibitor; MMF, mycophenolate mofetil; mTOR, mammalian target of rapamycin.

^e^Diabetes mellitus included existing disease and post-transplantation diabetes mellitus.

^f^CADI: chronic allograft damage index included interstitial inflammation, tubular atrophy, vascular intimal proliferation, interstitial fibrosis, mesangial matrix increase, and percentage of sclerotic glomeruli. The 6 components of CADI were graded semiquantitatively from 0 to 3 according to the Banff classification. (reference: Ortiz, F. *et al*. Predictors of renal allograft histologic damage progression. *J. Am. Soc. Nephrol*. **16**, 817–824 (2005)).

**Table 2:** Characteristics of renal transplant recipients with and without disease progression of kidney allograft.

Characteristic	Non-progressor	Progressor	P Value
	(n = 65)	(n = 31)	
Demographics			
Age at biopsy (yr)	49.7 ± 11.3	50.4 ± 13.5	0.784^a^
Men (n [%])	34 (52.3)	13 (41.9)	0.342^b^
Diabetes mellitus (n [%])	22 (33.8)	7 (22.6)	0.261^b^
Systolic BP (mmHg)	137.2 ± 21.9	137.0 ± 16.2	0.967^a^
Body mass index, (kg/m^2^)	23.8 ± 3.7	23.2 ± 4.9	0.443^a^
Timing of the biopsies, (months; median [interquartile range])	64.0 (2.6–157.6)	86.4 (23.2–137.5)	0.689^d^
Total HLA mismatches	3.1 ± 1.4	2.7 ± 1.2	0.138^a^
PRA class I ≥ 10% (n [%])	9 (13.8)	3 (9.7)	0.746^c^
PRA class II ≥ 10% (n [%])	10 (15.4)	2 (6.5)	0.326^c^
Previous acute rejection (n [%])	14 (21.5)	12 (38.7)	0.077^b^
Recipient risk score (A–D, 4 grades)	1.5 ± 0.6	1.6 ± 0.7	0.761^a^
Donor age (yr)	31.6 ± 10.8	30.3 ± 8.9	0.564^a^
Live donor (n [%])	19 (29.2)	9 (29.0)	0.984^b^
Cold ischemia time (hours)	8.7 ± 4.5	8.3 ± 4.0	0.691^a^
Donor risk score (0–39 points)	7.8 ± 6.2	7.6 ± 5.9	0.897^a^
High DcR3 expression (n [%])	22 (33.8)	22 (71.0)	0.001^b^
Laboratory data			
Serum albumin (g/dL)	3.8 ± 0.5	3.4 ± 0.6	0.001^a^
Total cholesterol (mg/dL)	198.5 ± 43.8	195.7 ± 64.9	0.808^a^
Urine protein (g/24 h; median [interquartile range])	0.46 (0.17–0.93)	1.49 (0.34–3.40)	0.004^d^
eGFR, MDRD (ml/min/1.73 m^2^)	34.0 ± 15.6	20.9 ± 10.8	<0.001^a^
Histopathology of renal allograft biopsy			
Acute rejection (n [%])	36 (55.4)	17 (54.8)	0.960^b^
TCMR	24 (36.9)	12 (38.7)	
ABMR	8 (12.3)	2 (6.4)	
Mixed TCMR and ABMR	4 (6.2)	3 (9.7)	
Borderline rejection (n [%])	17 (26.1)	7 (22.6)	0.705^b^
CAMR and Transplant glomerulopathy (n [%])	12 (18.5)	7 (22.6)	0.646^b^
IF/TA (n [%])			0.006^b^
0	20 (30.8)	5 (16.1)	
<25%	35 (53.8)	11 (35.5)	
26–50%	6 (9.2)	11 (35.5)	
>50%	4 (6.2)	4 (12.9)	
CADI scores (0–18 points)	5.3 ± 3.3	7.3 ± 3.3	0.008^a^
Banff tubulitis score	1.2 ± 1.0	1.5 ± 1.0	0.167^a^
Banff interstitial inflammation score	1.5 ± 0.9	1.8 ± 1.0	0.173^a^
Peritubular capillaritis score (median [interquartile range])^e^	0.0 (0.0–1.0)	0.0 (0.0–1.0)	0.225^d^
Glomerulitis score (median [interquartile range])	0.0 (0.0–1.0)	0.0 (0.0–1.0)	0.605^d^
C4d staining by IHC (C4d0 – C4d3) (median [interquartile range])^f^	0.0 (0.0–2.0)	0.0 (0.0–2.0)	0.561^d^
Intimal or transmural arteritis (median [min–max])	0.0 (0.0–2.0)	0.0 (0.0–3.0)	0.055^d^
Medications			
ACEI/ARB (n [%])	31 (47.7)	12 (38.7)	0.408^b^
Immunosuppression (n [%])			0.196^b^
CNI + MMF-based	57 (87.7)	24 (77.4)	
CNI + mTOR inhibitor-based	3 (4.6)	2 (6.5)	
mTOR inhibitor-based	0 (0.0)	2 (6.5)	
Other	5 (7.7)	3 (9.5)	

Comparison by

^a^independent-samples *t* test.

^b^Chi-square test.

^c^Fisher’s Exact test, and.

^d^Mann-Whitney test with IQR, interquartile range. C4d, Complement component 4d.

^e^Peritubular capillaritis score (reference: Gibson, I. W. *et al*. Peritubular capillaritis in renal allografts: prevalence, scoring system, reproducibility and clinicopathological correlates. *Am. J. Transplant*. **8**, 819–825 (2008)).

^f^C4d0 (negative); C4d1 (ATN-like minimal inflammation); C4d2 (Capillary and or glomerular inflammation (ptc/g >0) and/or thrombosis); C4d3 (Arterial – v3). (reference: Sis, B. *et al*. Banff ′09 meeting report: antibody mediated graft deterioration and implementation of Banff working groups. *Am. J. Transplant*. **10**, 464–471 (2010)).

**Table 3:** Association of baseline variables with kidney disease progression using multivariable Cox proportional hazard analysis.

Variable	model 1. adjusted for age and gender		model 2. adjusted for age, gender and other variables^a^	
	HR (95% CI)	P	HR (95% CI)	P
Diabetes mellitus	0.66 (0.28–1.56)	0.324		
Systolic blood pressure, per 10 mmHg increase	0.99 (0.82–1.18)	0.870		
Previous acute rejection	1.74 (0.84–3.61)	0.143		
High DcR3 expression	3.11 (1.42–6.78)	0.004	3.19 (1.40–7.27)	0.006
Serum albumin, 1 g/dl	0.24 (0.12–0.47)	<0.001	0.51 (0.22–1.22)	0.133
Proteinuria, 1 g/24 h	1.25 (1.09–1.42)	0.001	1.05 (0.85–1.29)	0.680
eGFR, per 10 ml/min/1.73 m^2^ increase	0.42 (0.29–0.61)	<0.001	0.48 (0.28–0.81)	0.006
Banff tubulitis score	1.34 (0.94–1.89)	0.099		
Banff interstitial inflammation score	1.38 (0.95–2.01)	0.088		
Peritubular capillaritis	0.65 (0.28–1.50)	0.317		
Intimal or transmural arteritis	2.30 (1.34–3.96)	0.003	1.48 (0.73–3.00)	0.272
C4d staining by IHC	1.61 (1.10–2.34)	0.010	1.33 (0.86–2.04)	0.201
IF/TA (26–50% + > 50%) *vs* (0 + < 25%)	3.61 (1.76–7.39)	<0.001	1.26 (0.40–4.02)	0.693
CADI	1.19 (1.06–1.34)	0.003	1.17 (0.99–1.38)	0.070

^a^The Cox proportional hazards model was used to evaluate the association of kidney disease progression with high DcR3expression in renal tubular epithelial cells, and the multi-variate analysis was adjusted for age, gender, serum albumin level, urine protein, estimated glomerular filtration rate, intimal or transmural arteritis, C4d staining by IHC, intensity of interstitial fibrosis and tubular atrophy (26–50% + > 50%) vs (0 + < 25%), and chronic allograft damage index (CADI). (Model 2)
