# Supplementary material for: Expression of decoy receptor 3 in kidneys is associated with allograft survival after kidney transplant rejection
Source: Sci Rep. 2015 Sep 3;5:12769. doi: 10.1038/srep12769 (PMC4558610; doi:10.1038/srep12769)

Expression of decoy receptor 3 in kidneys is associated with allograft survival after kidney transplant rejection

Shuo-Chun Weng[1,2,3], Kuo-Hsiung Shu[3,4], Ming-Ju Wu[1,3,4,5], Mei-Chin Wen[4,6], Shie-Liang Hsieh[1,7], Nien-Jung Chen[8,9,*] & Der-Cherng Tarng[1,9,10,11,*]

[1]Institute of Clinical Medicine, National Yang-Ming University, Taipei, Taiwan; [2]Center for Geriatrics and Gerontology, Taichung Veterans General Hospital, Taichung, Taiwan; [3]Division of Nephrology, Department of Internal Medicine, Taichung Veterans General Hospital, Taichung, Taiwan; [4]School of Medicine, Chung Shan Medical University, Taichung, Taiwan; [5]School of Medicine, College of Medicine, China Medical University, Taichung, Taiwan; [6]Department of Pathology, Taichung Veterans General Hospital, Taichung, Taiwan; [7]Genomics Research Center, Academia Sinica, Taipei, Taiwan; [8]Institute of Microbiology and Immunology, School of Life Sciences, National Yang-Ming University, Taipei, Taiwan; [9]Inflammation and Immunity Research Center, National Yang-Ming University, Taipei, Taiwan; [10]Department and Institute of Physiology, National Yang-Ming University, Taipei, Taiwan; [11]Division of Nephrology, Department of Medicine, Taipei Veterans General Hospital, Taipei, Taiwan.

[*]Correspondence and requests for materials should be addressed to: D.-C. T. (dctarng@vghtpe.gov.tw) and N.-J. C. (njchen@ms.ym.edu.tw)

[*]Current address: Department and Institute of Physiology, National Yang-Ming University, and Division of Nephrology, Department of Medicine, Taipei Veterans General Hospital, 201, Sec. 2, Shih-Pai Road, Taipei 11217, Taiwan. Tel:

+886-2-2826-7080 ; and Institute of Microbiology and Immunology, School of Life Sciences, National Yang-Ming University,155, Sec.2, Linong Street, Taipei, 112 Taiwan. Tel: +886-2-2826-7000-7106

Supplementary Information

Supplementary legends

Supplementary Figure S1. Decoy receptor 3 is also expressed in the medulla of kidney.

Supplementary Figure S2. Given that the HDE group showed more pronounced tubular injury than the LDE group, we tested whether DcR3 expression was increased (compared to controls without obvious acute kidney injury, AKI) in biopsies showing acute tubular injury without rejection or borderline infiltrates.

Supplementary Figure S3. Correlation of DcR3 molecule with acute allograft rejection.

Supplementary Figure S4. *In situ* hybridization (ISH) study and immunohistochemical (IHC) staining for different severity of kidney allografts.

Supplementary Figure S5. TNF-α-induced DcR3 release is dose-dependently increased in HK2 cells.

Supplementary Figure S6. Concordance was found with a positive correlation between high DcR3 expression in allograft tissue and high serum human serum enzyme-linked immunosorbent assay (ELISA) level.

Supplementary Table S1. Comparison of the response to routine therapy between low and high Decoy receptor 3 expression of repetitive kidney biopsies of allograft recipients.

Supplementary Figure S7. Calculation of quantitative immunohistochemical staining value by Image ProPlus (A-C).

Supplementary Figure S1. Decoy receptor 3 is also expressed in the medulla of kidney (B). A photograph of immunohistochemical staining for DcR3 in renal biopsy tissue. Scale bar = 50 μm.

A. Proximal renal tubules

B. Distal renal tubules in the medulla of kidney

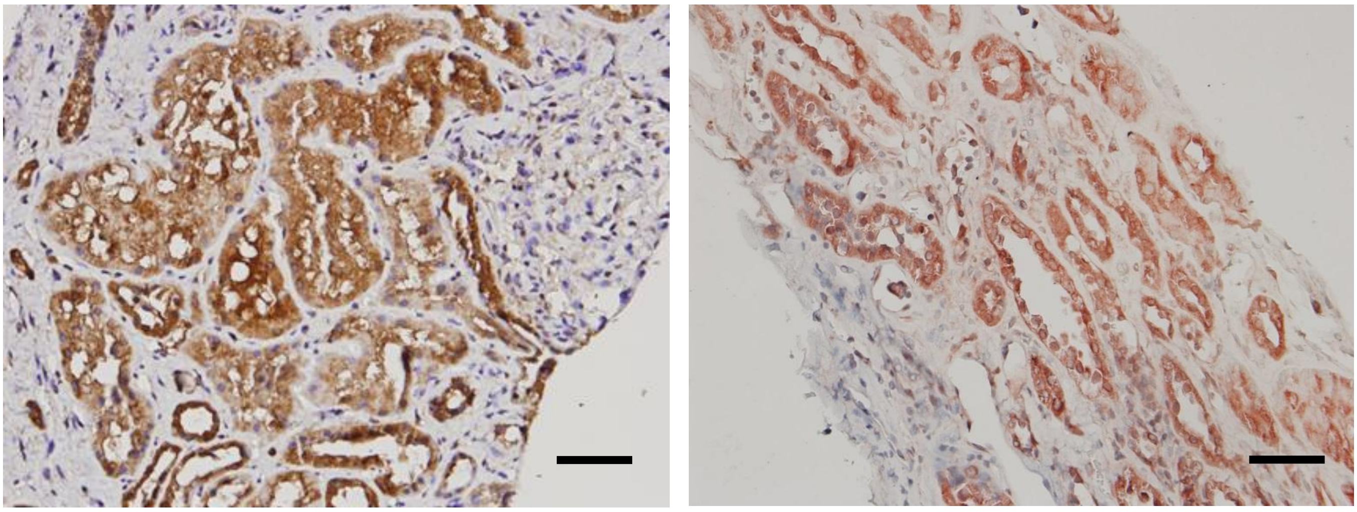

Supplementary Figure S2. Given that the HDE group showed more pronounced tubular injury than the LDE group, we tested whether DcR3 expression was increased (compared to controls without obvious acute kidney injury, AKI) in biopsies showing acute tubular injury without rejection or borderline infiltrates. (A) Although there may be some tubular injury in rejection kidney tissue, during organ procurement, and organ implantation, it is shown that the more severe kidney allograft rejection is, the more acute kidney injury, tubulitis, and interstitial mononuclear leukocyte infiltration are observed. (B) The others were computer-assisted quantitative immunohistochemical staining value (QISV) over four groups. (C) AKI scoring was based on the RIFLE criteria. (D, E, F) The pathological findings were analyzed by the renal pathologist, and they made decision by Banff 09 criteria. $^{*}P < 0.05$ HDE or LDE or borderline infiltrates *vs* no rejection. $^{\#}P < 0.05$ HDE or LDE *vs* borderline infiltrates. $^{\dagger}P < 0.05$ HDE *vs* LDE. Scale bar, 50μm.

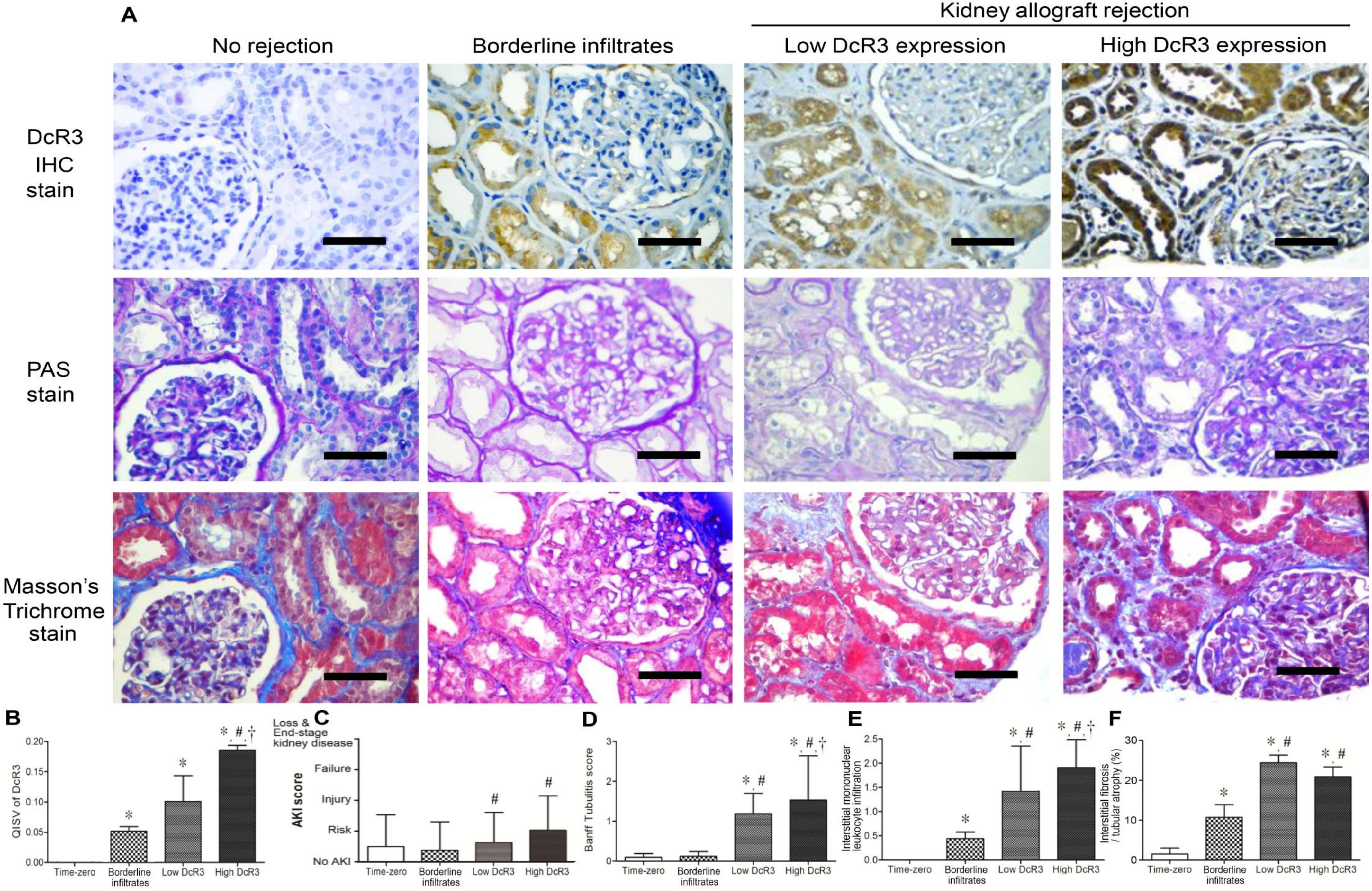

A
Kidney allograft rejection
No rejection
Borderline infiltrates
Low DcR3 expression
High DcR3 expression
DcR3 IHC stain
PAS stain
Masson's Trichrome stain
B
QISV of DcR3
Time-zero
Borderline infiltrates
Low DcR3
High DcR3
C
AKI score
Loss & End-stage kidney disease
Failure
Injury
Risk
No AKI
D
Banff Tubulitis score
E
Interstitial mononuclear leukocyte infiltration
F
Interstitial fibrosis / tubular atrophy (%)

Supplementary Figure S3. Correlation of DcR3 molecule with acute allograft rejection. (A) A high percentage of patients with high DcR3 expression (HDE) had more severe acute T cell-mediated rejection (TCMR). (B) There was no significant difference between HDE and LDE in different severities of acute antibody-mediated rejection (ABMR). (C) There was a positive correlation between HDE and tubulitis. (E) A positive correlation between HDE and interstitial mononuclear leukocyte infiltration, (D and F) but no direct relationship among HDE, peritubular capillaritis, and glomerulitis. (C-D) Data are expressed as means ± standard deviation.

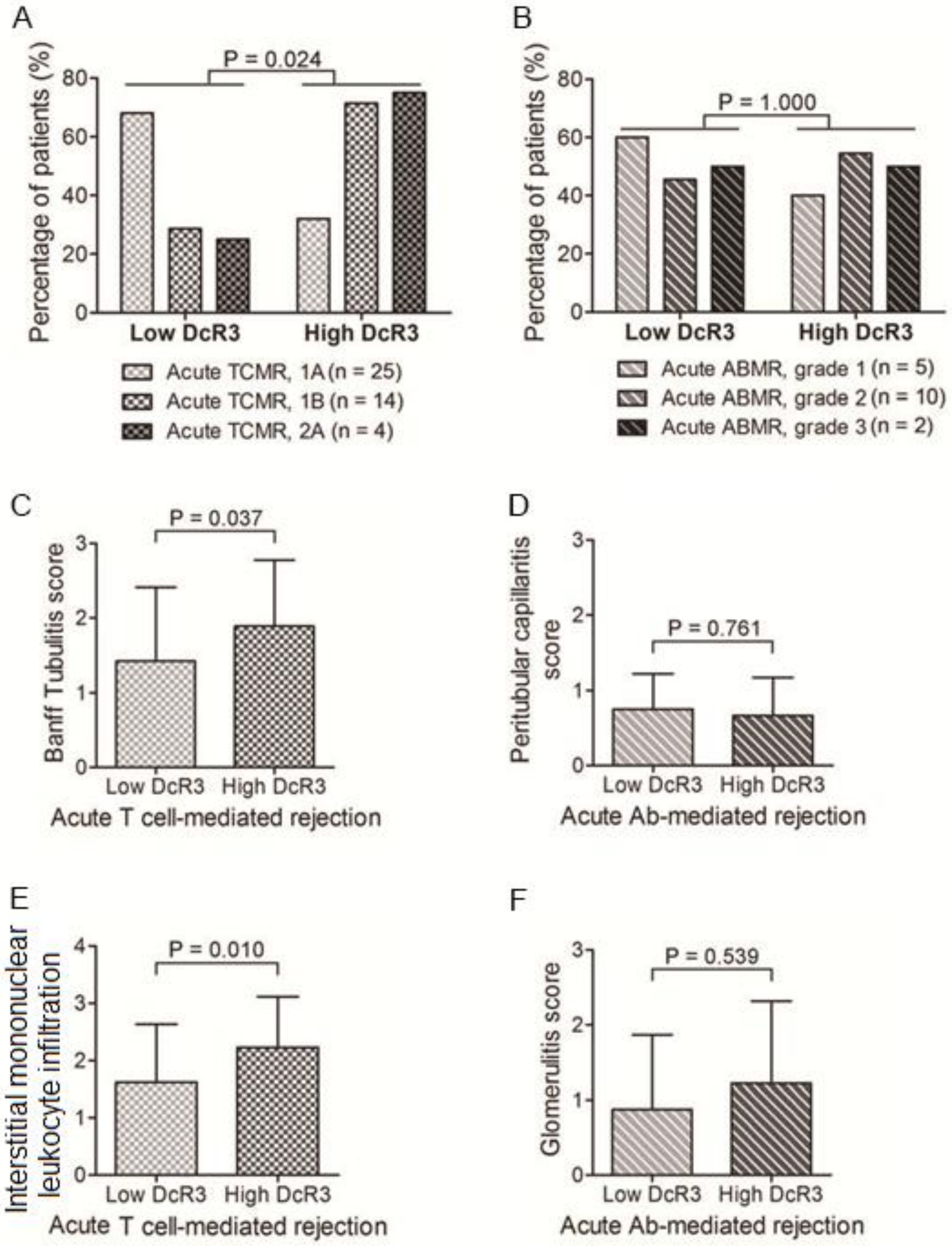
A
Percentage of patients (%)
P = 0.024
Low DcR3
High DcR3
Acute TCMR, 1A (n = 25)
Acute TCMR, 1B (n = 14)
Acute TCMR, 2A (n = 4)
B
Percentage of patients (%)
P = 1.000
Low DcR3
High DcR3
Acute ABMR, grade 1 (n = 5)
Acute ABMR, grade 2 (n = 10)
Acute ABMR, grade 3 (n = 2)
C
Banff Tubulitis score
P = 0.037
Low DcR3
High DcR3
Acute T cell-mediated rejection
D
Peritubular capillaritis score
P = 0.761
Low DcR3
High DcR3
Acute Ab-mediated rejection
E
Interstitial mononuclear leukocyte infiltration
P = 0.010
Low DcR3
High DcR3
Acute T cell-mediated rejection
F
Glomerulitis score
P = 0.539
Low DcR3
High DcR3
Acute Ab-mediated rejection

Supplementary Figure S4. ***In situ* hybridization (ISH) studies and immunohistochemical (IHC) staining for different severity of kidney allografts.** (A) different severity of kidney allograft by DcR3 IHC staining. (B) Dark blue tubular signals (white arrow) were considered positive for the presence of DcR3 mRNA. Scale bar, 50 μm.

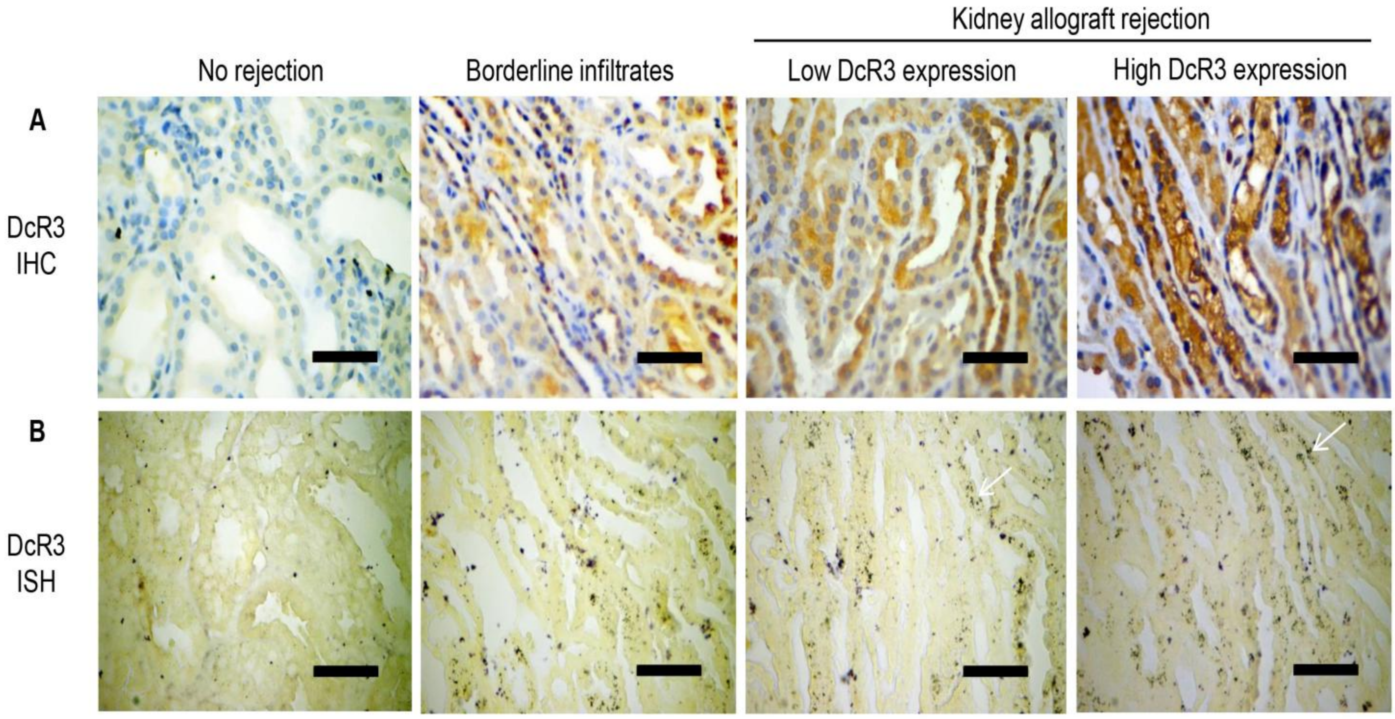

Supplementary Figure S5. TNF-α-induced DcR3 release is dose-dependently increased in HK2 cells.

For 24 hours, 24-well tissue culture plates containing stable HK2 cells were repetitively treated with sequential doses of TNF-α (0, 1, 5, 10 ng/ml). We used several inhibitors of cell mitosis to prove the supernatant was from the cell line. The induction of DcR3 was not suppressed by inhibition of the cell mitosis signaling pathway - p38 mitogen-activated protein kinase (MAPK), but it was suppressed by inhibitors of cell mitogen protein, such as c-Jun N-terminal kinase (JNK), extracellular signal-regulated kinase (ERK), and nuclear factor (NF)-κB. The procedure included both TNF-α (10 ng/ml) and a specific inhibitor for p38 MAPK (SB203580), either JNK (SP600125), ERK (PD98059) , or NF-κB (PDTC), with a titrating dose for co-treatment of the cell culture for 24 hours. Results are expressed as the means ± SD of two independent experiments performed in triplicate. Significance code: $^{*}P < 0.05$ and $^{**}P < 0.01$ indicates HK2 cells treated with TNF-α versus HK2 cells treated with TNF-α with increasing doses. $^{\dagger}P < 0.05$ indicates HK2 cells treated with TNF-α 10 ng/mL alone were co-treated with selective inhibitors for 24 hours, including p38 mitogen-activated protein kinase inhibitor SB203580 (Sigma Chemicals; St. Louis, MO) at 0.5, 1 and 2μ mol/L, c-Jun N-terminal kinase inhibitor SP600125 (Sigma Chemicals) at 5, 10 and 20 μmol/L, extracellular signal-regulated kinase inhibitor PD98059 (Sigma Chemicals) at 5, 10 and 20 μmol/L, and nuclear factor-κB inhibitor PDTC (Sigma Chemicals) at 25, 50 and 100 μmol/L (calculated by independent *t* test).

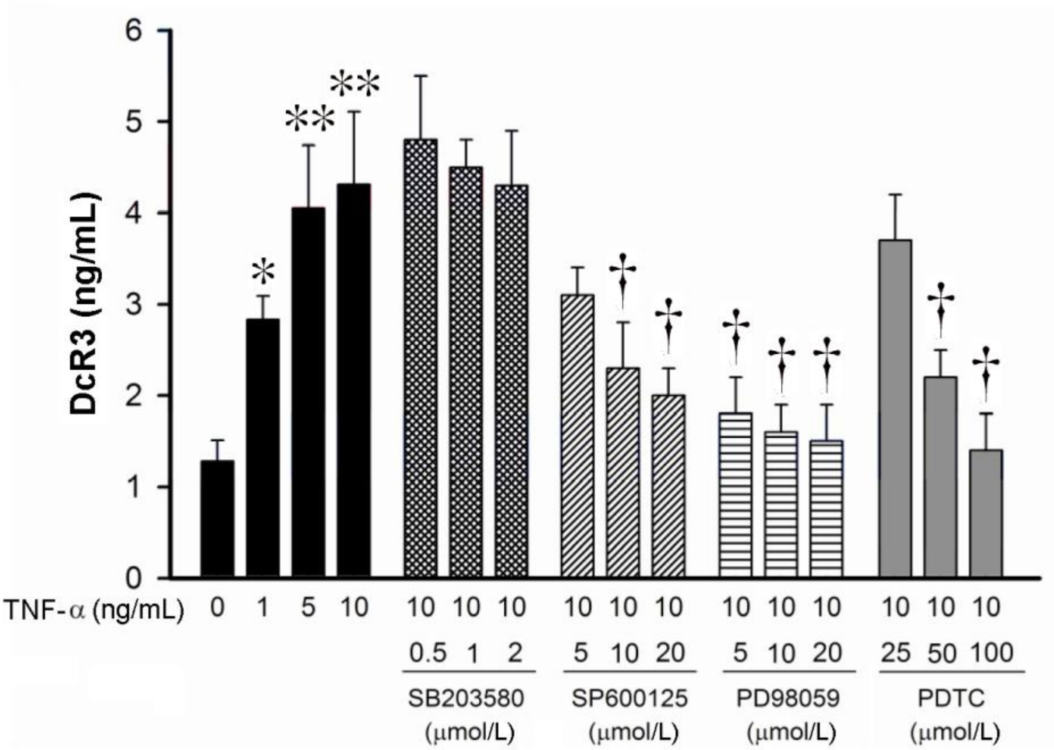

Supplementary Figure S5 Methods

***In Vitro* Study of Human Renal Tubular Cells**

Human papilloma virus(HPV)-16 transformed human proximal tubular epithelial cell line HK-2 cells were generously provided by Dr. C.-C.Hung (Chang Gung Memorial Hospital, Taiwan). Cells were cultured in Dulbecco's modified Eagle's medium/Ham's F12 (Gibco BRL, Paisley, UK) supplemented with 10% fetal bovine serum (FBS), glutamine, Hepes buffer (4-(2-hydroxyethyl)-1-piperazineethanesulfonic acid), hydrocortisone, insulin and sodium selenite (Sigma-Aldrich). All cell lines were grown at 37°C in 5% CO2.

After the culture medium was discarded, HK-2 cells were treated with 0.1, 1, and 10 ng/mL of TNF-α (R&D, Minneapolis, MN) for 24 hours. The levels of DcR3 in cell culture supernatant were determined by a DcR3 ELISA kit according to the manufacturer's instructions (BioLegend, LEGEND MAX™ ELISA Kit with

pre-coated plates, Cat. No. 438507, San Diego, CA 92121, R&D Center). The assay sensitivity was approximately 0.15 ng/mL. To further investigate the interrelationship between DcR3 and the TNF-α signaling pathway, HK2 cells were treated with TNF-α (10 ng/mL) in the presence of selective inhibitors for 24 hours, including SB203580 (Sigma Chemicals; St. Louis, MO) at 0.5, 1 and 2 μmol/L for p38 mitogen-activated protein kinase, SP600125 (Sigma Chemicals) at 5, 10 and 20 μmol/L for c-Jun N-terminal kinase, PD98059 (Sigma Chemicals) at 5, 10 and 20 μmol/L for extracellular signal-regulated kinase, and PDTC (Sigma Chemicals) at 25, 50 and 100 μmol/L for nuclear factor-κB.

Supplementary Figure S6. Concordance was found with a positive correlation between high DcR3 expression in allograft tissue and high serum human serum enzyme-linked immunosorbent assay (ELISA) level.

The human serum DcR3 concentration was not high enough to cope with the modulation of T-cell responses when compared with levels in mice treated with human DcR3-Fc or transgenic overexpression (150-850 ng/mL). The HDE group had high serum DcR3 levels (1.52 ± 0.36 ng/mL) compared with the levels (0.71 ± 0.27 ng/mL) of the LDE group ($P < 0.001$). (references: Ka, S. M., Sytwu, H. K., Chang, D. M., Hsieh, S. L., Tsai, P. Y. & Chen, A. Decoy receptor 3 ameliorates an autoimmune crescentic glomerulonephritis model in mice. *J. Am. Soc. Nephrol.* **18**, 2473–2485 (2007).; Chang, Y. C. *et al.* Epigenetic control of MHC class II expression in tumor-associated macrophages by decoy receptor 3. *Blood* **111**, 5054–5063 (2008).).

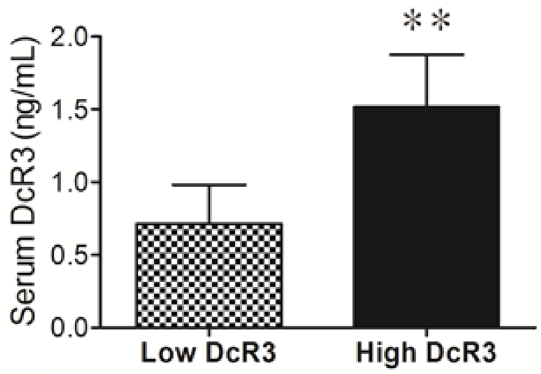

Supplementary Figure S6 Methods

All blood samples drawn from patients who had fasted over night were separated and kept frozen at –70°C when not analyzed immediately. Serum levels of DcR3 were measured using commercially available ELISA kits (BioLegend, LEGEND MAX^{TM} ELISA Kit with pre-coated plates, Cat. No. 438507, San Diego, CA 92121, R&D Center) according to the manufacturer’s instructions. Intra-assay and inter-assay coefficients of variation for DcR3 were 7.0% and 8.5% when loading 0.15 ng/mL, and

7.5% and 7.2% when loading 50 ng/mL, respectively.

Supplementary Table S1. Comparison of the response to routine therapy between low and high Decoy receptor 3 expression of repetitive kidney biopsies of allograft recipients.

Immunohistochemical staining	Methylprednisolone (MTP) pulse therapy after this rejection	Rejection episodes during these two-biopsy episodes	Increased dose of calcineurin inhibitor (CNI); mycophenolatemofetil (MMF); mammalian target of rapamycin (mTOR); prednisolone.					Response to anti-rejection therapy
DcR3 expression			Y/N	Prograft	MMF	Prednisolone	mTOR	
Low (case 1)	1 time	0	Yes	2mg BID	500mg BID →700mg BID →1000mg BID	20mg QD →5mg QD		Yes
Low (case2)	1 time	0	No	6mg BID	540mg BID →500mg BID	15mg QD →5mg QD		Yes
Low (case3)	1 time	1 (acute T-cell rejection)	No	6mg QD→5mg QD	250mg BID →250mg QD	0mg QD →5mg QD		No
Low (case 4)	0	1 (Ab-mediated rejection, plasmaphoresis)	No	2.5mg BID→1.5mg BID	540mg BID →540mg BID	10mg QD →5mg QD		Yes
Low (case 5)	1 time (and plasmaphoresis)	0	No	3mg BID→3mg BID	500mg BID →500mg BID	5mg QD →5mg QD		Yes
Low (case 6)	1 time (and anti-thymoglobulin	1 (acute T-cell rejection)	No	5mg QD→1.5mg BID	720mg BID →0mg	15mg BID →15mg		No

	therapy)					BID		
Low (case 7)	1 time (and anti-thymoglobulin therapy)	1 (acute T-cell rejection)	Yes	Cyclosporine 100mg QD, 50mg QN→Prograft 3mg BID	500mg BID →750mg BID	10mg QD →10mg QD	Certican 0.5mg BID	Yes
Low (case 8)	0	2 (Ab-mediated rejection,Rituximab, Methylprednisolone pulse therapy, plasmaphoresis)	Yes	3.5mg BID→4mg BID	500mg BID →0mg	10mg QD →0mg	Certican 1mg BID	No
Low (case 9)	0	1 (acute T-cell rejection)	No	3mg QD→1mg QD	750mg BID →250mg BID	5mg QD →5mg QD	Certican 1.5mg BID	Yes
Low (case 10)	0	1 (acute T-cell rejection)	No	5mg BID→2mg BID	1000mg BID →500mg BID	15mg QD →5mg QD	Certican 0.25mg QD	No
Low (case 11)	0	0	No	2mg BID→2mg BID	500mg BID →500mg BID	10mg QD →5mg QD		Yes
Low (case 12)	0	1 (acute T-cell rejection)	No	9mg BID→5mg BID	750mg BID →500mg BID	15mg QD →5mg QD		Yes
Low (case 13)	0	0	No	3.5mg BID→3mg QD	540mg BID →180mg BID	10mg QD →5mg QD		Yes
Low (case 14)	0	0	No	Cyclosporine 175mg BID→100mg BID	1000mg BID →750mg BID	15mg BID →0mg		Yes

High (case 1)	1 time	2 (Acute T cell and Ab-mediated rejection, Methylprednisolone pulse therapy x2, plasmaphoresis)	No	5mg BID→2.5mg BID	0mg→0mg	10mg QD →0mg		Yes
High (case 2)	0	0	No		750mg BID →500mg BID	10mg QD →5mg QD	Sirolimus 1mg Q3D →1mg Q3D	No
High (case 3)	1 time	3 (Ab-mediated rejection,Methylprednisolone pulse therapy x3, plasmaphoresis)	Yes	2.5mg BID→2.5mg BID	540mg BID →1000mg BID	5mg QD →0mg QD		No
High (case 4)	1 time	0	No	6mg QD→0mg	540mg BID →0mg	5mg QD →5mg QD	Sirolimus 1mg QD	Yes
High (case 5)	2 times	2 (Ab-mediated rejection,Methylprednisolone pulse therapy x1, plasmaphoresis x 2)	Yes	1mg BID→2mg BID	180mg BID →500mg BID	5mg QD →0mg		No
High (case 6)	2 times	3 (Acute T cell and Ab-mediated rejection,Methylprednisolone pulse therapy x2, Rituximab x1, plasmaphoresis x 1)	No	6mg BID→0mg	500mg BID →0mg		Certican 1mg BID	No
High (case 7)	0	0	Yes	3mg QD→3mg QD	250mg TID →250mg BID	10mg QD	Sirolimus 1mg QD	No
High (case 8)	1 (refuse MTP pulse)	0	Yes	Cyclosporine 50mg BID→50mg BID	180mg TID →360mg BID	15mg QD →10mg QD		No

High (case 9)	1 time	1 (Ab-mediated rejection,Methylprednisolone pulse therapy x1, plasmaphoresis x 1)	Yes	Cyclosporine 50mg BID→50mg BID		5mg QD →30mg QD		Yes
High (case 10)	1 time	1 (acute T-cell rejection)	No	Cyclosporine 50mg BID→50mg BID				Yes
High (case 11)	0	1 (acute T-cell rejection)	Yes	Cyclosporine 100mg BID→Prograft 2mg BID	500mg BID →500mg BID	10mg QD →5mg QD	Certican 0.5mg BID	Yes
High (case 12)	0 (refuse MTP pulse)	1 (Ab-mediated rejection)	No	5mg BID→3mg BID	1000mg BID →1000mg BID	30mg QD →5mg QD		Yes
High (case 13)	1 time	2 (acute T-cell rejection)	No	Prograft 2.5mg BID→Cyclosporine 125mg BID	500mg BID →0mg	5mg QD →20mg QD		No
High (case 14)	1 time	0	Yes	4mg BID→9mg QD	750mg BID →750mg BID	20mg QD →30mg QD		Yes

Supplementary Figure S7. Calculation of quantitative immunohistochemical staining value by Image ProPlus (A-C). Positive Decoy receptor 3 (DcR3) immunostaining in cells was chosen as the color of interest (hue 0–35, saturation 30–255, intensity 0–255 for DcR3 segmentation) and masked in bright red color by Image Pro Plus software. Counterstaining of hematoxylin was chosen as the background area (hue 100–255, saturation 0–255, and intensity 0–255 for hematoxylin segmentation) and masked in green. Integrated optical density of DcR3 was calculated as pixel intensity of DcR3 (bright red) multiplied by their pixel area. Quantitative immunohistochemical staining value of DcR3 was then calculated by the integrated optical density of DcR3 divided by the total sum of DcR3 (bright red, black arrow) and hematoxylin (green, blue arrow) staining area. Area of tubular lumen, interstitial fibrosis and inflammatory cell infiltrate were not included for quantification. (B) The evaluated cells and (C) interstitium were calculated separately.

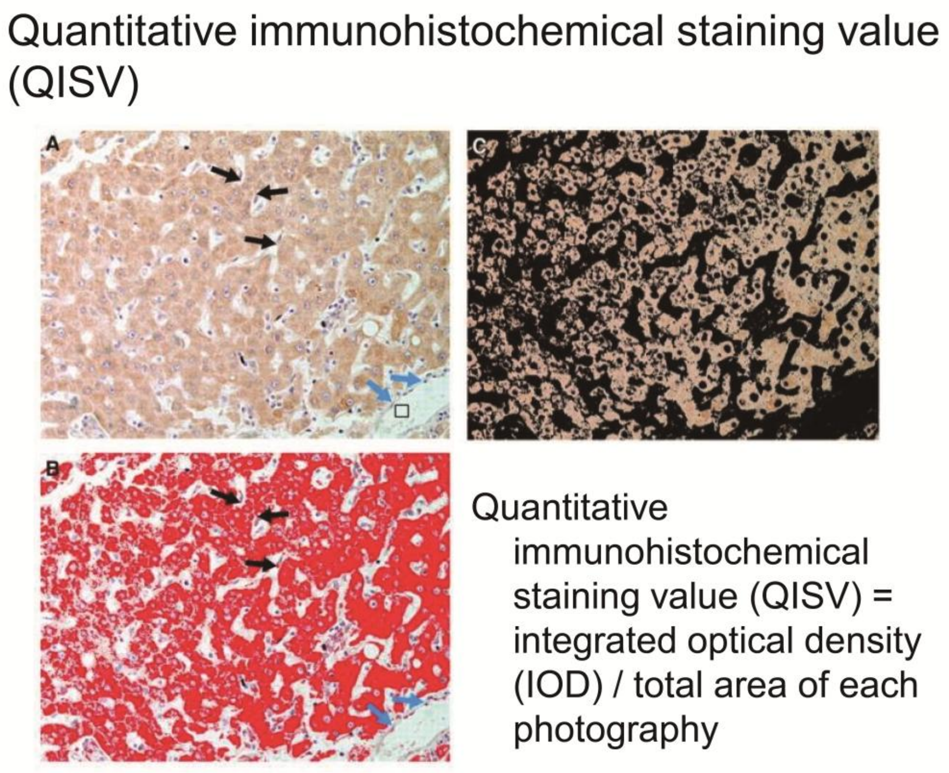

Supplement: Supplementary Information [file srep12769-s1.pdf]
